# K-Carrageenan/Locust Bean Gum Gels for Food Applications—A Critical Study on Potential Alternatives to Animal-Based Gelatin

**DOI:** 10.3390/foods13162575

**Published:** 2024-08-17

**Authors:** Simona Russo Spena, Rossana Pasquino, Nino Grizzuti

**Affiliations:** DICMaPI—Dipartimento di Ingegneria Chimica dei Materiali e della Produzione Industriale, Università degli Studi di Napoli Federico II, P.le V. Tecchio 80, 80125 Naples, Italy; simona.russospena@unina.it (S.R.S.);

**Keywords:** vegetable hydrocolloids, gelatin, texture, rheology

## Abstract

Among hydrocolloids used in the food industry, gelatin (an animal protein) is remarkably known for its unique gel forming ability. Creating a perfect, green substitute for animal gelatin is extremely difficult if not impossible, because this versatile hydrocolloid offers many special properties that are not easily imitated by other vegetable-based systems. The combination of more than one type of hydrocolloid is commonly used in food either to bridge the above-mentioned gap or to impart novel organoleptic characteristics (such as mouthfeel) to food products, to modify rheological characteristics, and to satisfy processing requirements in the industry. In this work, we study the rheology and the texture of water mixtures of κ-Carrageenan (κ-C) and Locust Bean Gum (LBG). By fixing different κ-C concentrations and varying the LBG/κ-C ratio, we explore a wide range of potentially useful textures. The results obtained for the green systems are also compared to those exhibited by animal gelatin formulations.

## 1. Introduction

Among the commercial hydrocolloids used in the food industry, Animal Gelatin (AG) is remarkably known for its unique gel-forming ability, serving multiple functions and a wide range of applications in various industries. Gelatin is a biopolymer solution composed of proteins, mineral salts, and water [1]. The polymer derives from the partial hydrolysis of collagen molecules, which are available in animal connective tissues.

The replacement of gelatin by vegetal substitutes has been an issue for many years in the vegetarian, halal, and kosher markets, and has gained increased interest in the last decade. The application of green hydrocolloids is considered an integral part of food industries. The market demand was estimated at USD 8.8 billion in 2018 and is predicted to grow at a compound annual growth rate of 5.3% from 2018 to 2025, to reach USD 11.4 billion by 2025 [2,3,4,5]. AG offers many special properties that are not easily imitated by other hydrocolloids, particularly (i) thermally reversible gelling properties around body temperature, a quality known as “melt-in-mouth”, which gives gelatin unique organoleptic properties and flavor release; (ii) an appropriate gel strength and elasticity; and (iii) pH stability.

Creating a perfect substitute for animal gelatin is extremely difficult, if not impossible. According to Morrison et al. [6], the strategy to creating substitutes for foodstuffs should be application/process-specific. Focusing on soft-solid, gel-based foods, their texture depends on the gelling capabilities of polysaccharides and proteins, the two main families of polymers that are used as texture modifiers [7,8,9]. Several examples can be found in the literature. Tang et al., for example [7], studied the effects of Locust Bean Gum and κ-Carrageenan on sodium caseinate. Specifically, they showed that the addition of LBG implied a strong gel behavior while κ-Carrageenan determined a soft gel. The combination of more than one type of hydrocolloid is commonly used in food formulations to impart novel organoleptic properties (mouthfeel), to modify rheological characteristics, and to satisfy industrial processing requirements [10,11,12].

Gelled systems are often described by means of rheological quantities. Linear viscoelasticity is widely used, often by measuring the elastic and loss modulus as a function of both oscillation frequency and amplitude, thus characterizing the type and strength of the gel [13,14,15]. Temperature ramps are also very popular as they can detect the sol–gel or gel–sol transitions of hydrocolloids. Rheology, however, due to its instrumental nature, provides a somewhat indirect description of the material texture and of how such a texture affects the sensorial perception of food. It is important to underline that building rheology–sensory relationships can be difficult due to the food complex composition and structure, as well as to its changes during oral processing. Over time, much work has been carried out to develop mechanical tests that try to imitate or substitute the sensory evaluation of food texture [16]. One technique providing this type of information is Texture Profile Analysis (TPA). First developed in the 1960s [17,18,19], TPA introduced rating scales of sensorial evaluation, thus setting the basis of texture profile methodology [20]. The latter allows for the quantitative description of several texture characteristics in a variety of food products.

Numerous attempts have been made to mimic the texture of gelatin-based gels by combining different, non-animal gelling agents. Among them, κ-Carrageenan (κ-C) has always been considered [21,22]. κ-C is derived from a family of linear polysaccharides extracted from red algae. It easily dissolves in warm water, forming a random coil. Upon cooling, it undergoes a conformational transition from coils to aggregated double helices. This results in a gel structure stabilized by hydrogen bonds. For the above reasons, κ-C has great potential as a gelling agent to produce vegan products. Pure κ-C gels, however, are fragile, easy to fracture, and only moderately elastic. To improve the gel quality, and to better simulate the animal gelatin behavior, κ-C is often associated with other polysaccharides.

Locust Bean Gum (LBG) is a high-molecular-weight, non-ionic galactomannan polysaccharide, extracted from the seeds of Ceratonia Siliqua. It is composed of β-(1-4)-mannose backbones, randomly branched by α-(1-6)-galactose. It is mainly used as an additive (E410) in the food and beverage industry, most often as a thickening, stabilizing, and gelling agent, or emulsifier [23]. LBG in water can form gels only in the presence of other compounds, for example, at high sugar concentrations or in combination with other hydrocolloids such as κ-C. The addition of LBG to κ-C has several consequences. The mixtures may form gels under conditions where pure κ-C does not. Furthermore, LBG has a synergistic effect on the strength of κ-C gels and makes them more cohesive and more elastic. Its high ability to hold water limits syneresis.

The scope of this work is twofold. On the one hand, we evaluated the gel performance of the system composed of κ-C with the addition of different amounts of LBG. These two polysaccharides were purposedly chosen as they are considered a potential base to produce jelly confectionery products. This was performed by correlating the rheological response to the textural analysis. On the other hand, by taking the κ-C/LBG gel as a case study, we compared its behavior to that of the animal-based gelatin counterpart. The comparison, along with a careful analysis of the available literature (which is discussed in the Results section) suggests that vegetal-based gels possess rheological, textural, and sensorial attributes that place them in a somewhat different window of performance with respect to the animal-gelatin-based formulations.

## 2. Materials and Methods

### 2.1. Materials

κ-C and LBG were provided by Sigma-Aldrich (Baden-Württemberg, Germany). Based on manufacturer’s specifications, κ-C and LBG present molecular weights, respectively, of 1.1 × 10^5^ g/mol and 9.7 × 10^5^ g/mol. Viscosity values provided at 0.3% wt, 100 s^−1^, and 25 °C for κ-C and at 1% wt, 100 s^−1^, and 25 °C for LBG are, respectively, 5–25 mPa and 2300–5000 mPa. Pig skin gelatin (275 Bloom grade) was kindly supplied by the Perfetti van Melle company (Lainate, Italy). For gelatin pig skin, no further information is available, as it is subject to confidential agreement. Hydrocolloids were used as received. Solutions of various LBG/κ-C mass ratios (1:2, 1:4, 1:6, and 1:10) and κ-C contents (1, 1.5 and 2 wt%) were prepared by dispersing the hydrocolloids in bi-distilled water for 1 h by magnetic stirring at room temperature. The temperature was then raised to 80 °C and the system was kept under agitation for 1 additional hour to guarantee complete dissolution. Each sample was labeled as “κ-C x/y:z”, where x represents the κ-C concentration and y:z represents the mass ratio between LBG and κ-C. The “κ-C x” label refers to the pure κ-C solutions.

Using the same protocol, but a different temperature (60 °C), a pig skin gelatin sample at a weight concentration of 6.67% was prepared. This concentration is generally used as a standard reference [24]. All solutions were stored at 4 °C and, before each test, were kept in a closed vessel at the test temperature for 30 min to erase any thermal history.

The choice of the system and of the experimental variables was made after careful considerations of various aspects. First, mixtures of κ-C and LBG were used because we are presently considering them as a potential base formulation for the production of jelly confectionary products, which are mostly based on animal gelatin formulations. We chose a range of κ-C concentrations that were able to get as close as possible to the properties of the gelatin standard (6.67 wt%, 275 Bloom grade). Finally, LBG was added, at a fixed κ-C concentration, to improve the quality of the gel. The maximum LBG concentration was set by the observation (see the Results section) where, above a saturation value, the system properties were not improved.

### 2.2. Rheology

Rheological properties were measured by a rotational-stress-controlled rheometer (Anton Paar MCR-702, Graz, Austria) equipped with 25 mm parallel plates at a 1 mm gap. Temperature was controlled by a Peltier unit with a stability of ±0.1 °C. A solvent trap and a low-viscosity oil seal were used to prevent sample evaporation. To avoid wall slip, possibly caused by water loss, the plates were covered with sandblasted paper with a roughness of 10.3 ± 0.8 μm.

The following rheological protocol was applied to all samples. They were loaded in the sol state at 80 °C and cooled down to 20 °C at a rate of 5 °C/min. Oscillatory shear flow at a strain of 1% (below the linear viscoelastic limit) and a frequency of 10 rad/s was applied to monitor the viscoelastic evolution across the sol–gel transition. The obtained gel properties were then evaluated by a test sequence composed of a frequency sweep test at a 1% strain, an oscillatory time sweep test at the frequency of 10 rad/s and 1% amplitude, and an amplitude sweep test at the same frequency of 10 rad/s.

### 2.3. Texture Profile Analysis Procedure

In the standard Texture Profile Analysis (TPA), two consecutive cycles of compressive deformation, separated by a resting time zone, are applied to the gel and the resulting force is measured as a function of time. TPA was performed on a universal uniaxial testing machine, a CTX Texture Analyzer (AMETEK Brookfield, Middleboro, MA, USA). The force was measured by a load cell of 10 N at full scale. Measurements were performed with a crosshead equipped with a cylindrical probe of 40 mm in diameter. A 0.05 N load threshold was used to determine the initial contact between the tool and the sample. During the test, the crosshead moves at 3 mm/s downwards, compressing the sample until a deformation of 0.5 is reached, i.e., 50% of its original height. The loading process takes a time of about one second, which has been chosen as a characteristic time for a standard bite. When the maximum deformation is reached, the movement is inverted, keeping the same speed and bringing the head back to its original position. The cycle is followed by a pause of 1 s, and a second cycle repeats the pattern of the first one. TPA was performed six times on each system, using each time a fresh sample.

**Figure 1:**
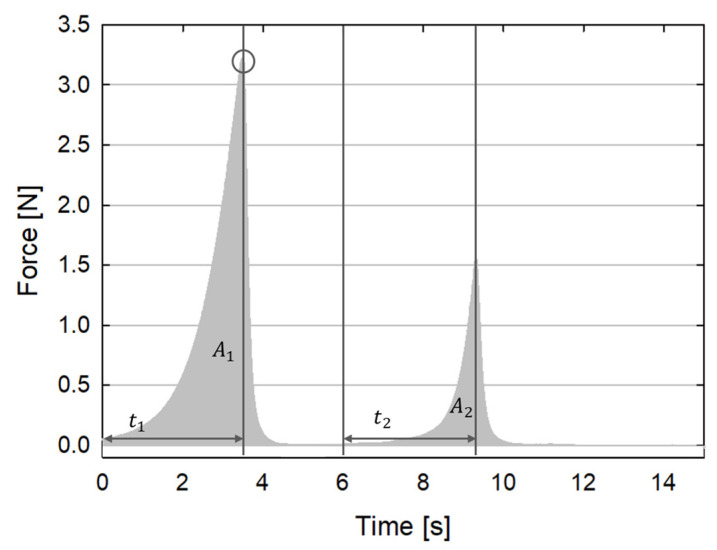
Representative TPA curve (force as a function of time) for sample κ-C 1/1:6.

The typical outcome of a TPA experiment is reported in Figure 1 for one of the samples studied in this work. Numerous parameters can be obtained by analyzing the TPA response. Here, only the following primary parameters were taken into consideration:

- Hardness, obtained by measuring the peak load reached during the first deformation cycle (the black circle in Figure 1). Hardness is related to the stiffness of the material.
- Springiness, given by the ratio between the time interval needed to reach the peak load since the start of the second cycle and the same time interval for the first cycle (t_2_/t_1_ in Figure 1). Springiness is related to the recovery of the material and to its viscoelastic properties.
- Cohesiveness, given by the ratio between the area under the time/force curve during the second cycle divided by the same area during the first cycle (A_2_/A_1_ in Figure 1). Cohesiveness is related to the consistency of the material. Its value varies from 0 to 1. The larger its value, the more the material keeps its texture unaltered.

## 3. Results

### 3.1. Thermo-Reversibility: Melt-in-Mouth Requirements

**Figure 2:**
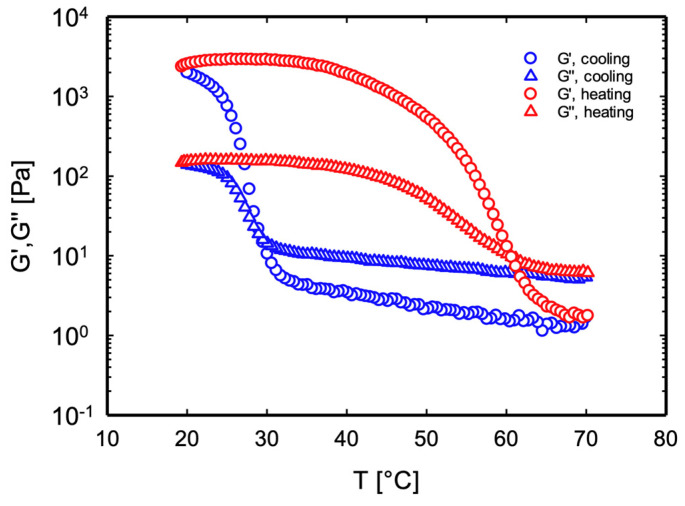
The loss (G″) and elastic (G′) moduli as a function of temperature during cooling and heating ramps at 5 °C/min for sample κ-C 1.5/1:10.

To analyze the melt-in-mouth requirements, all samples were subjected to cooling/heating dynamic ramp tests at a rate of 5 °C/min. By way of an example, the results for sample κ-C 1.5/1:10 are reported in Figure 2. For all other samples, the response was qualitatively similar (see Supplementary Materials). During cooling, the moduli in the liquid phase (G″ < G′) modestly increase, until a sharp change and a crossover are observed in correspondence with the sol–gel transition. Symmetrically, upon heating, a first gradual and then sharper decrease is measured, corresponding to the inverse gel–sol transition. As often reported in the literature [25,26], the temperatures marking the crossover, respectively, T_SOL-GEL_ and T_GEL-SOL_, are chosen as the fingerprint of the transition. Table 1 shows the values of T_SOL-GEL_ and T_GEL-SOL_ for all samples.

Table 1 shows that upon increasing κ-C concentration, as well as the amount of LBG to a fixed κ-C content, both T_SOL-GEL_ and T_GEL-SOL_ increase. The relevant parameter in the evaluation of the melt-in-mouth property is obviously T_GEL-SOL._ The latter spans from, ca., 43 °C to, ca., 58 °C for pure κ-C. The addition of LBG determines a further increase, up to a maximum value of about 70 °C for κ-C 2/1:1.

Having already mentioned the gelling mechanism of pure κ-C solutions in the Introduction, we add that the most accepted model for gelation of the κ-C/LGB mixtures postulates interaction between the helical structure of the carrageenan and the unsubstituted mannan region of the galactomannan chains [27,28,29,30]. In this way, a continuous network is generated by the binding of the sparsely unsubstituted regions along galactomannan molecules onto double helices. Because of the extra, mixed-junction zones, it is not surprising that less carrageenan double-helix content is required for gelation to occur in this case. The gelling process, however, is still driven by the random coil to double-helix transition of κ-C. As the concentration of LBG increases, both transition temperatures increase, due to the above-described interaction between the helical structure of the carrageenan and the unsubstituted mannan region of galactomannan chains.

### 3.2. Gel Elasticity: Shear Moduli at 20 °C

**Figure 3:**
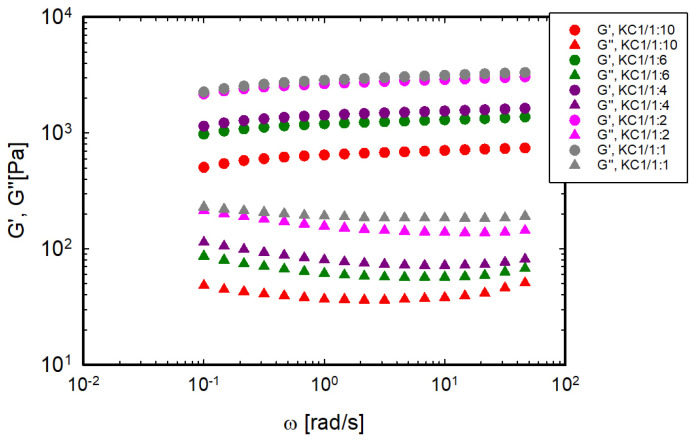
The loss (G″) and elastic (G′) moduli of the κ-C 1%/x: y gels as a function of frequency at 20 °C.

The storage and loss moduli of the gels at 20 °C are shown in Figure 3 as a function of frequency for the system based on 1% κ-C and various LBG:κ-C ratios. As expected, the moduli are almost independent of frequency, indicating the formation of a solid-like gel.

**Figure 4:**
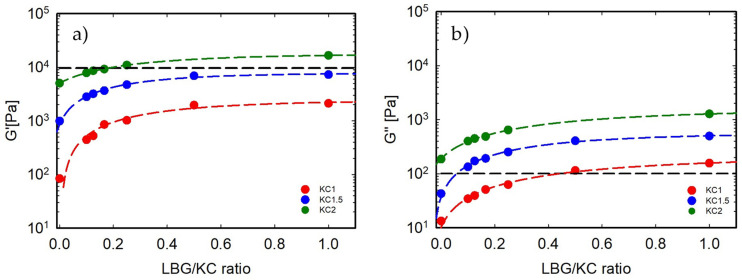
The elastic (**a**) and loss (**b**) moduli of the gels at 20 °C as a function of the LBG/κ-C ratio. The broken black line represents the value of the gelatin-based system.

Once the gel was formed, its stability was probed by an oscillatory time sweep test at 20 °C. The complete set of results, reported in Supplementary Materials, indicates that the moduli of all samples remain essentially constant. Their mean values are plotted in Figure 4a,b. It is apparent that once the κ-C concentration is fixed, an increase in LBG content strengthens both the elastic and the loss components.

The results shown in Figure 4 agree with those obtained by Tako et al. [29], who studied the mechanical properties of κ-C/LBG blends. Although their experimental conditions are somewhat different, they also found that at high LBG concentrations, the elastic modulus levels off. This behavior has been explained by considering that the interactions between κ-C and LBG chains consist of the formation of a carrageenan–galactomannan complex. At low galactomannan concentrations, the elastic modulus increases due to intermolecular binding. At high LBG concentrations, however, all galactomannan sites are saturated, and a further concentration increase does not affect the blend modulus. This behavior has been explained by a more dispersed network compared to that obtained for the same number of κ-C chains but a smaller number of galactomannan units [30,31].

### 3.3. Non-Linear Response of the Gel

**Figure 5:**
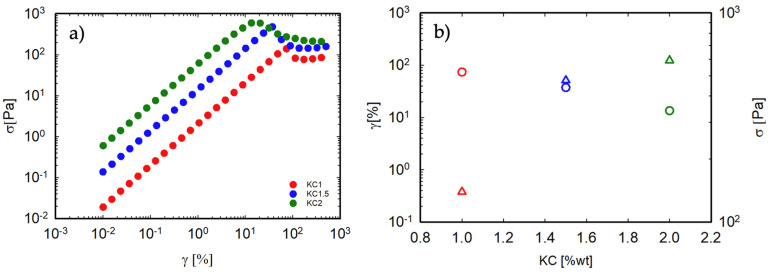
(**a**) Oscillatory shear stress as a function of strain for pure κ-C gels at 20 °C. The oscillation frequency is 10 rad/s. (**b**) Stress (triangles) and strain (circles) at break as a function of κ-C concentration as derived from (**a**). Color code as in (**a**).

The non-linear properties of a food gel are particularly relevant when its ultimate performance is considered, for example, when the so-called “first bite” effect is considered. To gain better insight into this point, strain sweep tests were performed on the gels. The oscillatory stress as a function of strain is shown in Figure 5a for the pure κ-C gels. At low strains, as expected, the stress increases linearly with strain, up to a limiting value, beyond which the gel network breaks down. The stress at break is plotted as a function of the corresponding strain in Figure 5b. As reported in [32], increasing the κ-C concentration determines an increase in the stress at break and a decrease in the corresponding strain, as the correlation length becomes smaller and, therefore, the network becomes stiffer and more fragile. These results represent one main weakness of κ-C gels. As the concentration increases, a progressively stronger gel is obtained, but this is accompanied by an increase in brittleness. Notice that, by doubling the concentration from 1% to 2%, the strain at break decreases by about an order of magnitude.

**Figure 6:**
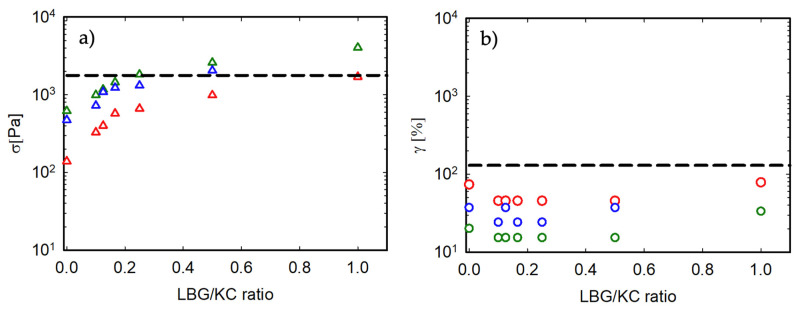
(**a**) Stress and (**b**) strain at break as a function of the LBG/κ-C ratio for the gels at 20 °C. The oscillation frequency is 10 rad/s. Color code is as in Figure 5, indicating different κ-C concentrations. The broken black line is the response of the animal gelatin reference.

Figure 6 summarizes the values of stress and strain at break for all samples. At a fixed κ-C concentration, the addition of LBG generates an increase in the stress at break. On the contrary, the breaking strain remains roughly unchanged. This means that by adding LBG, it is possible to increase the stress at break of the gels without increasing their brittleness.

### 3.4. Comparison with Animal Gelatin

As mentioned in the Introduction, within the commercial hydrocolloids used in the food industry, AG is known for its superior gel properties, and its replacement by vegetal substitutes is a challenging task. AG is also particularly well performing as far as “melt-in-mouth” and gel strength are concerned. Having tested the gel performance of a green hydrocolloid (κ-C) and its synergic behavior with LBG over a wide range of compositions, it is natural to attempt a comparison with an AG-based gel formulation. The comparison is made with the 6.67%wt AG system because such a concentration is widely used in the candy industry as a reference formulation.

Table 1 informs that AG has a T_GEL-SOL_ of about 37 °C. This parameter is strictly dependent upon gelatin concentration but varies only from 35 to 40 °C in a range of AG concentrations of 6–10% [33]. Without going into the details of the transition mechanism, it is well known that the melting temperature of gelatin is directly related to the shrinkage of the source collagen and that this depends upon the primary sequence of the collagen which, in turn, depends upon its source [34].

All κ-C/LBG mixtures studied here present a T_GEL-SOL_ substantially higher than that of AG and well above the body temperature. We believe that this behavior can be extended to most vegetable hydrocolloids, especially those based on polysaccharides, as confirmed by several literature examples. In a recent work, Wu et al. [32] studied the viscoelastic properties of a gel based on Konjac Glucomannan and κ-C with the addition of maltodextrins. As in the present work, the sol–gel and gel–sol temperatures were measured at the moduli crossover during cooling/heating ramps. All samples in [32] displayed a T_GEL-SOL_ higher than 40 °C. This also agrees well with previous work from our group, where κ-C/Konjac Glucomannan samples were characterized [23]. Other examples include mixtures based on κ-C and Xanthan gum. Avallone et al. [33], for example, showed that by adding Xanthan gum (in a range of 0–1.5%) to a κ-C-based sample, T_GEL-SOL_ ranges between 44 and 50 °C. Many other examples can be reported, including gellan gum, carboxymethylcellulose [35], agar–agar [36,37], and more. All these polymers share some common features: (i) they belong to a polysaccharide class; (ii) they undergo thermo-reversible gelation; and (iii) their T_GEL-SOL_ is always substantially higher than that of animal gelatin. Regarding the latter point, the only possibility to match (or get close) to the AG performance is to use the lowest possible concentration required for gel formation. Doing that, however, undermines the mechanical properties of the final gel. To summarize, the melting temperature issue, closely related to the melt-in-mouth effect, seems to currently be an impassable limitation for vegetable jelly systems as compared to animal gelatin. Possible alternatives for the future might consider even more complex formulations, such as double physical carbohydrate gels, combinations of carbohydrates with vegetal proteins, and the chemical modification of already used hydrocolloids. A few attempts have already been made, although with poor results, like the deacetylation of Konjac Glucomannan [38,39].

Moving to the mechanical aspects, the main strength of animal gelatin is elasticity. The AG sample chosen here as a reference presents $G^{\prime }\approx 10^{4}\;Pa$ and $G^{\prime \prime }\approx 45\;Pa$. G′ is a measure of the resistance of the material to being deformed elastically when a stress is applied. It can be somewhat related to the consistency of the gel and, as a consequence, to its hardness. G″, in turn, represents the viscous (dissipative) portion of the viscoelastic behavior and can be related to the chewability of the sample. Several vegetable-based formulations do match one of the two values reported above. None of the mixtures studied here, however, can match the two values simultaneously, as clearly shown in Figure 4. If G′ were the only relevant parameter, the best κ-C/LGB systems would be κ-C 1.5/1:2 and κ-C 2/1:6. If, however, we tried to match G′’ to the corresponding AG value, the only sample falling close to AG would be κ-C 1/1:8. In no way, however, could we find a mixture matching both viscoelastic parameters to those of gelatin.

**Figure 7:**
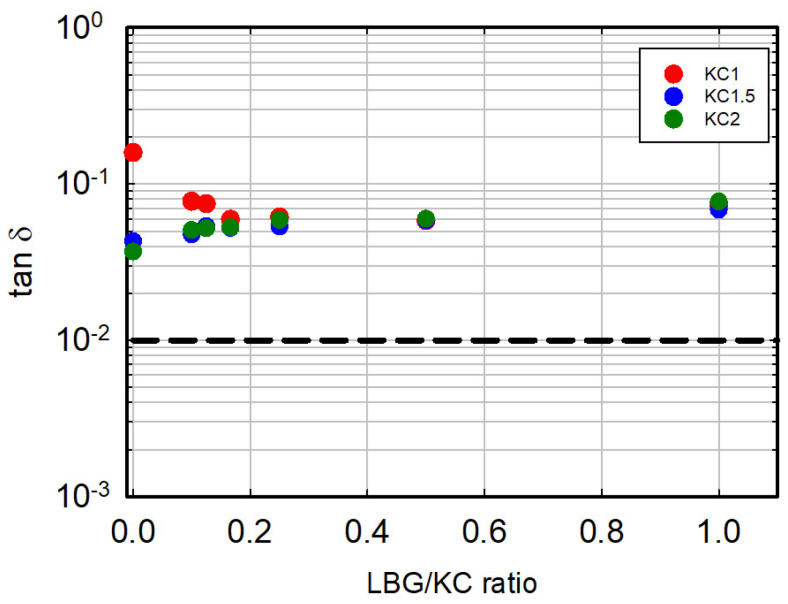
Tan δ of the LBG/κ-C system as a function of the LBG/κ-C ratio. Color codes are as in the previous figures. The broken black line is the tan δ value of the reference animal gelatin.

Strictly related to the modulus issue, an important parameter to be considered in the evaluation of the gel performance is the ratio between the loss and elastic moduli, known as tan δ. Its values, summarized in Figure 7, remain essentially around 0.1 for all formulations. On the contrary, the animal gelatin reference shows a value of about 0.01. The great dominance of the elastic component over the viscous contribution represents, in our opinion, the main feature and uniqueness of animal gelatin. For the vegetal-based systems, a tan δ value of about 0.1 is a peculiarity and is very distant from that displayed by animal gelatin.

To summarize, at least for the systems under consideration and considering the linear viscoelastic performance, none of the proposed combinations can simulate the AG reference. Animal gels have, at least from this point of view, quite different properties.

To conclude this section, it must be underlined that, as now expected, the non-linear behavior of the two families of gels is also quantitatively very different. This can be appreciated in Figure 6a,b, where the broken black lines represent the stress and strain at break for the animal gelatin. Their values are, respectively, 1780 Pa and 130%. Also, in this case, none of the studied, green-based mixtures are able to simultaneously match these two values. A stress at break similar to that of AG might be achieved by κ-C 1/1:1, κ-C 2/1:4, and κ-C 1.5/1:2. The strain at break, however, even with the addition of LBG, remains roughly the same as that of the pure κ-C samples and significantly lower than that of gelatin.

### 3.5. Texture Profile Analysis

The textural primary parameters of the gels are given in Table 2. They refer to the set of characteristics that determines the so-called “fist bite” sensory perception. Hardness is used to evaluate the sensation of food in the mouth and represents the force that must be applied to bring out a given, pre-determined deformation. As for the viscoelastic moduli and for the stress at break, hardness increases as the LBG concentration increases. However, focusing on the systems with an elastic modulus comparable to that of gelatin (e.g., κ-C 1.5/1:2 and κ-C 2/1:6), it clearly appears that their hardness is much larger than that of gelatin. Conversely, samples presenting a hardness comparable to animal gelatin are those with the lowest κ-C concentration (1%).

Springiness gives an indication of how long it takes for the material to recover its shape and properties after compression. Considering only the pure κ-C systems, as the κ-C concentration increases, springiness also increases. The same trend is determined by the addition of LBG. The animal gelatin reference, however, shows a springiness of 94%, a value that none of the vegetable samples can get close to. It is important to note that TPA values are strongly dependent on the imposed measuring parameters. Particularly, springiness is strictly dependent on the waiting time between the two consecutive compressions. In this case, as already specified above, the waiting time of 1 s was chosen in an attempt to mimic the characteristic time lapse between bites.

Cohesiveness probably showed the largest difference between the green-based samples and the animal gelatin counterpart. AG has a cohesiveness of about 88%, significantly higher than that exhibited by the vegetable systems. For the latter, the addition of LBG determines an increase in cohesiveness in a range of 30–55%. Cohesiveness represents the mechanical textural attribute related to the degree of deformation of the food before break. In sensory tests, performed by trained panelists, this quantity is evaluated through a compression of the sample between molars, measuring the amount of deformation before rupture [40,41,42,43]. A low cohesiveness implies a reduction in the amount of chewing required before the product can be swallowed. The measured values, translated into sensorial information, indicate that vegetable-based products are more easily broken between the teeth, compared to the animal gelatin sample, which requires longer chewing times.

## 4. Conclusions

Creating an ideal, green substitute for animal gelatin is extremely difficult. AG offers many special properties that cannot be easily imitated. In an attempt to fill this gap, in this study, vegetal-based gels were characterized, providing evidence for utilizing mixtures of hydrocolloids to tune the texture. The properties of systems based on κ-C and on its mixtures with LBG have also been compared to those of animal gelatin.

The effect of LBG addition on κ-C gels has been evaluated to underline the main similarities and/or differences between vegetable polysaccharides and animal-gelatin-based systems. The results show that the addition of LBG to κ-C effectively improves the gel performance, both in terms of brittleness and gel strength. The improved κ-C/LBG gels provide a wide range of textures, which can be exploited for the development of vegan gelled food as functional and sustainable ingredients in food industries. The data analysis has been conducted to understand a possible relation between measured variables and sensory evaluation. When compared to animal gelatin, however, vegan-based systems seem to present some impassable limitations, which appear to be a common feature to many vegetable-based hydrocolloids.

## Figures and Tables

**Table 1:** Sol–gel and gel–sol temperatures of pure κ-C and κ-C/LBG mixtures. The values for animal gelatin are also reported for comparison.

Sample	T_SOL-GEL_ [°C]	T_GEL-SOL_ [°C]	Sample	T_SOL-GEL_ [°C]	T_GEL-SOL_ [°C]	Sample	T_SOL-GEL_ [°C]	T_GEL-SOL_ [°C]
κ-C 1	21.8	43.0	κ-C 1.5	27.5	52.2	κ-C 2	32.3	58.1
κ-C 1/1:10	24.2	55.2	κC1.5/1:10	28.4	60.6	κ-C 2/1:10	34.0	64.8
κ-C 1/1:6	24.4	56.2	κ-C 1.5/1:6	29.2	60.6	κ-C 2/1:6	34.2	65.9
κ-C 1/1:4	25.6	56.4	κ-C 1.5/1:4	30.3	61.7	κ-C 2/1:4	35.3	66.5
κ-C 1/1:2	26.1	56.7	κ-C 1.5/1:2	30.2	61.5	κ-C 2/1:2	35.8	66.7
κ-C 1/1:1 AG	26.9 16.3	56.7 29.5	κ-C 1.5/1:1	35.6	65.4	κ-C 2/1:1	38.4	69.2

**Table 2:** Texture Profile Analysis parameters evaluated at 20 °C for all κ-C/LBG gels. The animal gelatin reference is added for comparison.

Sample	Hardness [N]	Springiness [%]	Cohesiveness [%]
κ-C 1	Too weak gel	Too weak gel	Too weak gel
κ-C 1/1:10	1.1	33%	15%
κ-C 1/1:6	2.9	48%	26%
κ-C 1/1:4	2.7	48%	32%
κ-C 1/1:2	3	53%	43%
κ-C 1/1:1	4.8	75%	48%
κ-C 1.5	3	50%	30%
κ-C 1.5/1:10	4.8	63%	33%
κ-C 1.5/1:6	5.23	68%	39%
κ-C 1.5/1:4	6.3	71%	40%
κ-C 1.5/1:2	6.5	80%	46%
κ-C 1.5/1:1	7	84%	58%
κ-C 2	7.5	75%	39%
κ-C 2/1:10	8.2	78%	39.56%
κ-C 2/1:6	9	78%	41.8%
κ-C 2/1:4	9.3	83%	46%
κ-C 2/1:2	10.2	85%	48.36%
κ-C 2/1:1	11	87%	54.7%
AG	3	94%	88%

## Data Availability

The original contributions presented in this study are included in this article, and further inquiries can be directed to the corresponding author.

## Supplementary Materials

The following supporting information can be downloaded at https://www.mdpi.com/article/10.3390/foods13162575/s1, Figure S1: The loss and elastic moduli as a function of temperature during cooling (a) and heating (b) ramps at 5 °C/min; Figure S2: The loss (b) and elastic (a) moduli evolution over time at 20 °C; Figure S3: The loss and elastic moduli as a function of angular frequency at 20 °C; Figure S4: The loss and elastic moduli as a function of temperature during cooling (a) and heating (b) ramps at 5 °C/min; Figure S5: The loss (b) and elastic (a) moduli evolution over time at 20 °C; Figure S6: The loss and elastic moduli as a function of angular frequency at 20 °C; Figure S7: The loss and elastic moduli as a function of temperature during cooling (a) and heating (b) ramps at 5 °C/min; Figure S8: The loss (b) and elastic (a) moduli evolution over time at 20 °C; Figure S9: The loss and elastic moduli as a function of angular frequency at 20 °C.
