# Supplementary material for: K-Carrageenan/Locust Bean Gum Gels for Food Applications—A Critical Study on Potential Alternatives to Animal-Based Gelatin
Source: Foods. 2024 Aug 17;13(16):2575. doi: 10.3390/foods13162575 (PMC11353981; doi:10.3390/foods13162575)
Supplement: Supplementary file 1 [file foods-13-02575-s001.zip › foods-3134076-supplementary.pdf]

Supplementary Information

- **KC1/x:y**

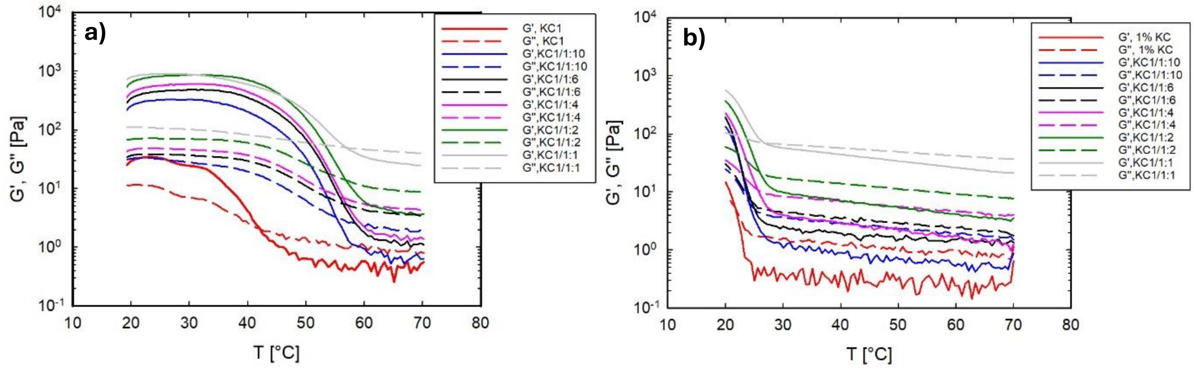

Figure S1. The loss and elastic moduli as a function of temperature during cooling (a) and heating (b) ramps at 5°C/min.

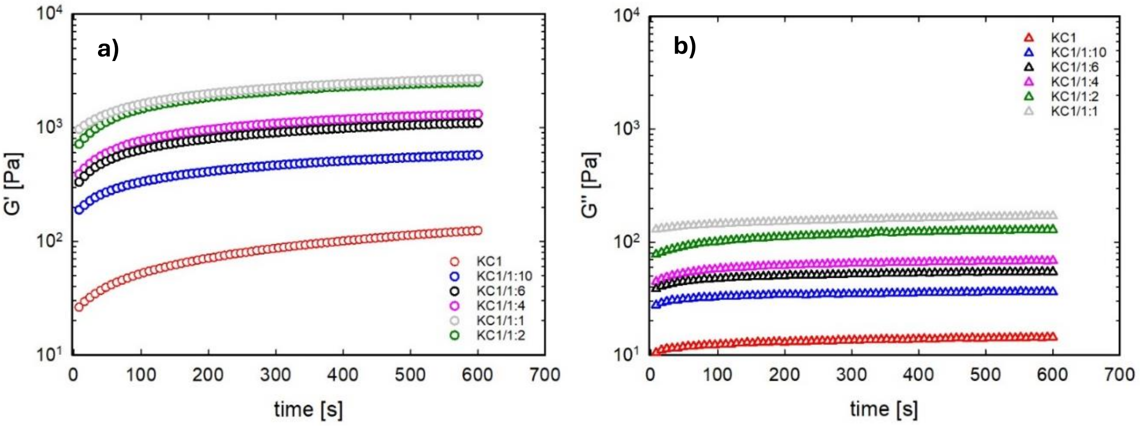

Figure S2. The loss (b) and elastic (a) moduli evolution over time at 20°C.

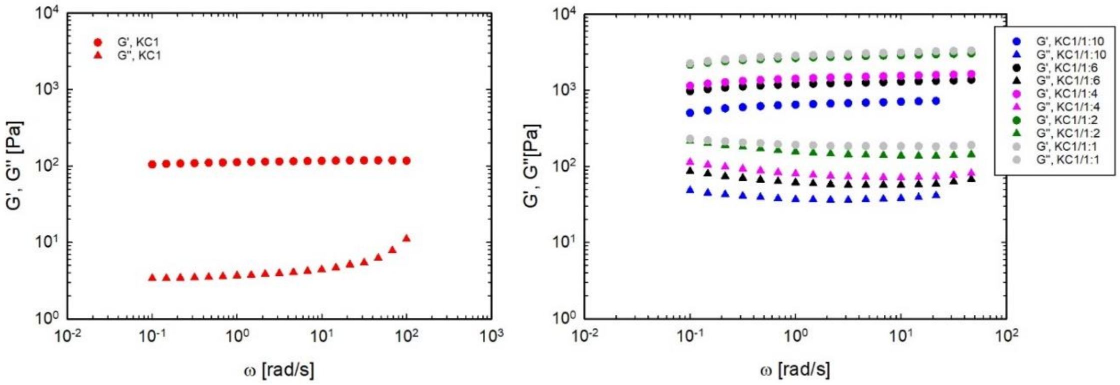

Figure S3. The loss and elastic moduli as a function of angular frequency at 20°C.

- **KC1.5/x:y**

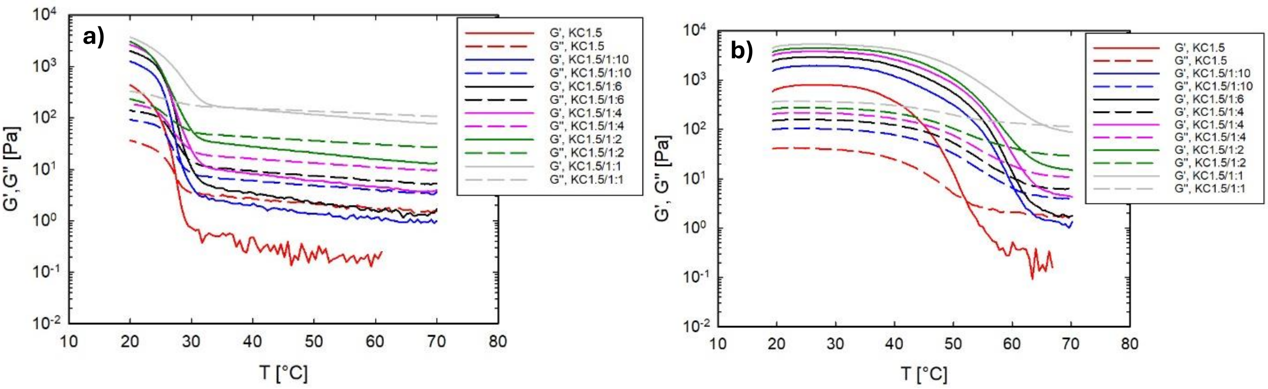

Figure S4. The loss and elastic moduli as a function of temperature during cooling (a) and heating (b) ramps at 5°C/min.

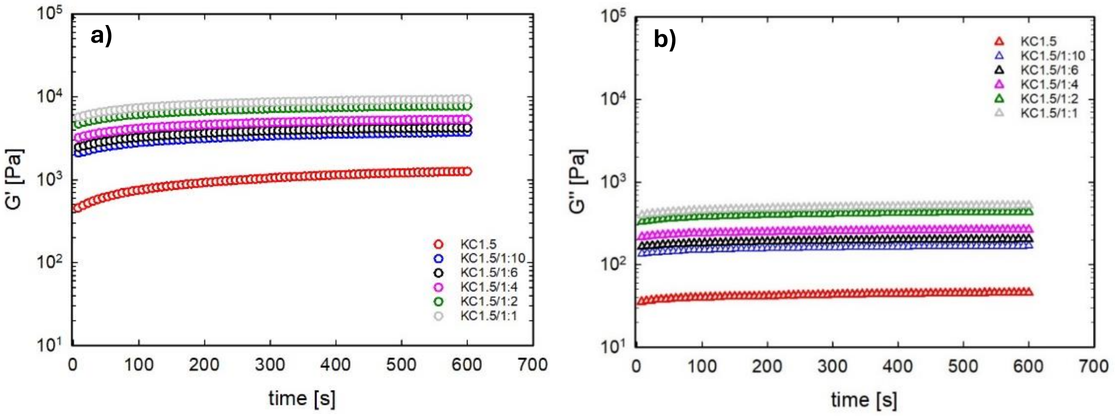

Figure S5. The loss (b) and elastic (a) moduli evolution over time at 20°C.

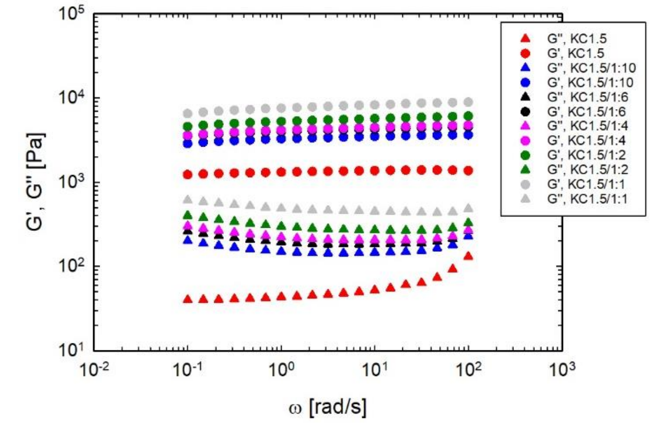

Figure S6. Elastic and loss moduli as a function of angular frequency at 20°C.

- **KC2/x:y**

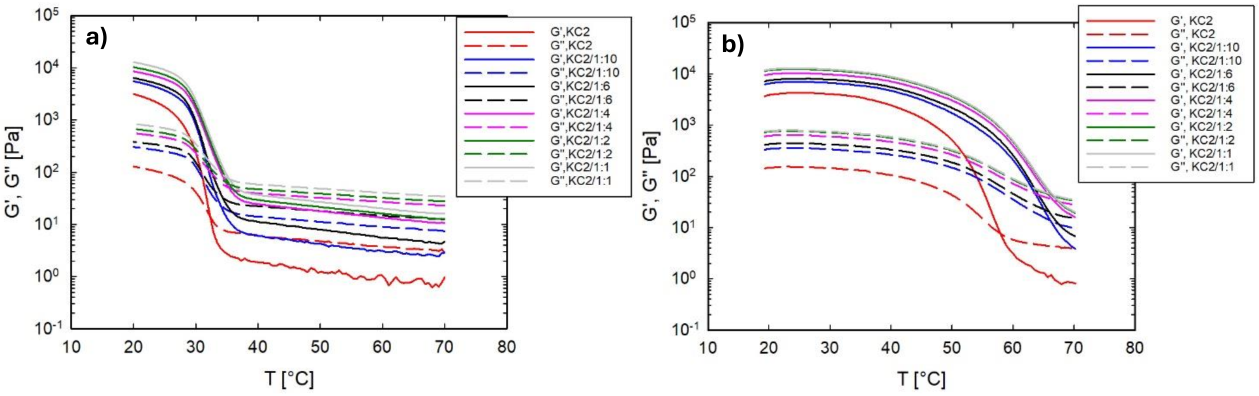

Figure S7. The loss and elastic moduli as a function of temperature during cooling (a) and heating (b) ramps at 5°C/min.

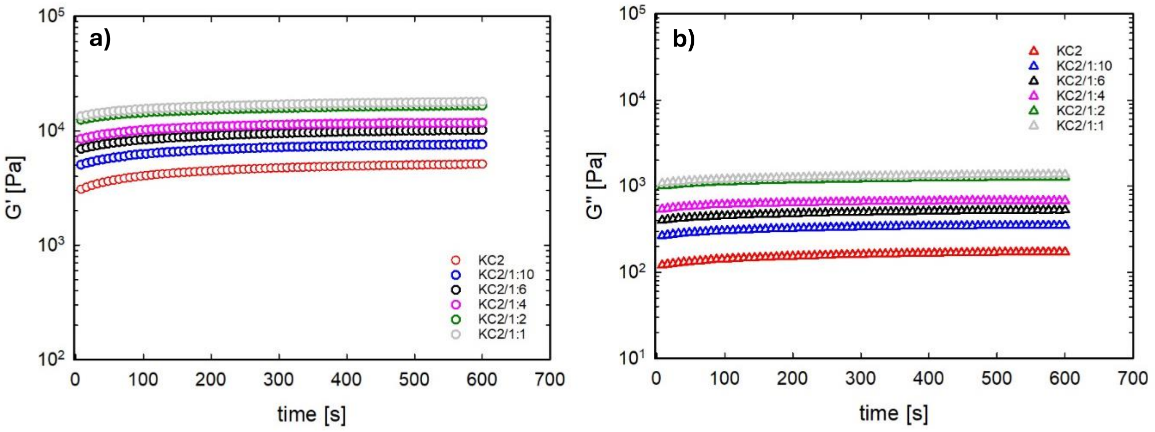

Figure S8. The loss (b) and elastic (a) moduli evolution over time at 20°C.

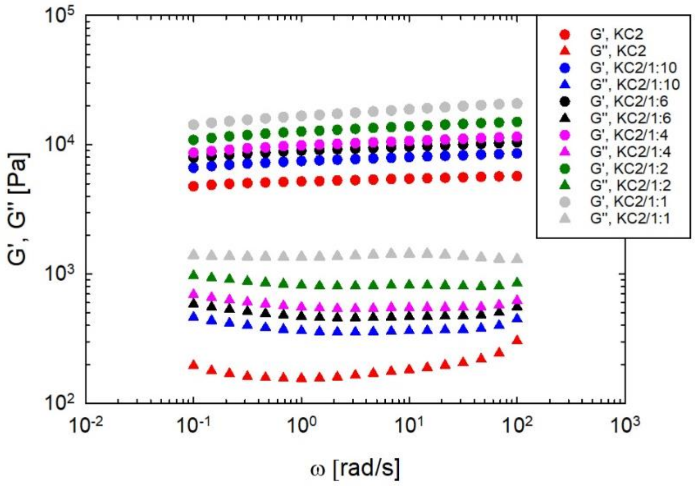

Figure S9. The loss and elastic moduli as a function of angular frequency at 20°C.
